# Developing a PmSLP3-based vaccine formulation that provides robust long-lasting protection against hemorrhagic septicemia–causing serogroup B and E strains of *Pasteurella multocida* in cattle

**DOI:** 10.3389/fimmu.2024.1392681

**Published:** 2024-05-21

**Authors:** Jamie E. Fegan, Regula C. Waeckerlin, Liyuwork Tesfaw, Epshita A. Islam, Getaw Deresse, Dawit Dufera, Eyob Assefa, Wubet Woldemedhin, Abinet Legesse, Mirtneh Akalu, Berecha Bayissa, Quynh Huong Nguyen, Dixon Ng, Sang Kyun Ahn, Anthony B. Schryvers, Takele A. Tefera, Trevor F. Moraes, Scott D. Gray-Owen

**Affiliations:** ^1^ Department of Molecular Genetics, Temerty Faculty of Medicine, University of Toronto, Toronto, ON, Canada; ^2^ Department of Microbiology, Immunology, and Infectious Diseases, Cumming School of Medicine, University of Calgary, Calgary, AB, Canada; ^3^ Department of Veterinary Bacteriology, National Veterinary Institute, Bishoftu/Debre Zeyit, Ethiopia; ^4^ Department of Biochemistry, Temerty Faculty of Medicine, University of Toronto, Toronto, ON, Canada

**Keywords:** gram-negative, *Pasteurella multocida*, hemorrhagic septicemia, vaccine, surface lipoprotein

## Abstract

**Background:**

*Pasteurella multocida* is a bacterial pathogen that causes a variety of infections across diverse animal species, with one of the most devastating associated diseases being hemorrhagic septicemia. Outbreaks of hemorrhagic septicemia in cattle and buffaloes are marked by rapid progression and high mortality. These infections have particularly harmful socio-economic impacts on small holder farmers in Africa and Asia who are heavily reliant on a small number of animals kept as a means of subsistence for milk and draft power purposes. A novel vaccine target, PmSLP-3, has been identified on the surface of hemorrhagic septicemia–associated strains of *P. multocida* and was previously shown to elicit robust protection in cattle against lethal challenge with a serogroup B strain.

**Methods:**

Here, we further investigate the protective efficacy of this surface lipoprotein, including evaluating the immunogenicity and protection upon formulation with a variety of adjuvants in both mice and cattle.

**Results:**

PmSLP-3 formulated with Montanide ISA 61 elicited the highest level of serum and mucosal IgG, elicited long-lasting serum antibodies, and was fully protective against serogroup B challenge. Studies were then performed to identify the minimum number of doses required and the needed protein quantity to maintain protection. Duration studies were performed in cattle, demonstrating sustained serum IgG titres for 3 years after two doses of vaccine and full protection against lethal serogroup B challenge at 7 months after a single vaccine dose. Finally, a serogroup E challenge study was performed, demonstrating that PmSLP-3 vaccine can provide protection against challenge by the two serogroups responsible for hemorrhagic septicemia.

**Conclusion:**

Together, these data indicate that PmSLP-3 formulated with Montanide ISA 61 is an immunogenic and protective vaccine against hemorrhagic septicemia-causing *P. multocida* strains in cattle.

## Introduction

*Pasteurella multocida* is a gram-negative bacterial pathogen that is able to infect a wide spectrum of wild and domestic animal hosts and leads to a variety of clinical presentations. *P. multocida* resides as a commensal in the upper respiratory tract of mammals and birds, and strains are classified into five capsular serogroups (serogroups A, B, D, E, and F) based on capsular polysaccharide profiles (1, 2). Bovine-associated *P. multocida* infections are mainly associated with two distinct diseases, bovine respiratory disease (BRD) and hemorrhagic septicemia (HS). BRD is a major concern for livestock in North America and Europe and is characterized by serogroup A and D strains of *P. multocida* that asymptomatically colonize the upper respiratory tract and can become pathogenic in situations of high stress, such as during transport or mixing of unfamiliar animals in a feedlot, hence its common name “shipping fever” [reviewed by Callan and Garry (3)]. BRD-associated strains of *P. multocida* are present worldwide, where the disease complex is often referred to as “pneumonic pasteurellosis.”

In sub-Saharan Africa and regions of South Asia, B and E serogroup strains of *P. multocida* present in a much more acute form as HS in cattle and buffaloes (4). Occasional outbreaks of HS are reported in Western countries in cattle and pigs (5), including the first reported HS outbreak in cattle recently in Spain (6); however, serogroup B and E strains are not considered endemic in Europe or North America. HS is characterized by seasonal outbreaks at several year intervals with a rapid onset and high mortality and has devastating effects on local livestock farmers at both an economic and cultural level (4, 7). Transmission generally occurs by direct contact with carrier animals that have survived an infection with HS and consistently carry bacteria in the tonsillar crypts and inconsistently in other compartments of the respiratory and gastrointestinal tracts (8). Attempts have been made to estimate economic losses from HS outbreaks in India (9) and Pakistan (10), but it remains difficult to determine exact incidence, distribution, and economic impact of HS on a country level in lower- and middle-income countries (LMICs) as the collection and reporting of outbreak data is largely left to national governments.

Vaccines against both bovine-associated *P. multocida* diseases are currently on the market, but they are primarily limited to aluminum-adjuvanted whole killed bacteria, known as bacterins (11), or live attenuated strains (12, 13), both of which offer serogroup specific protection and can be highly reactogenic. Especially in the case of HS vaccines, bacterin vaccines are mostly produced locally in LMICs and are adapted to currently circulating strains, which makes standardization, large-scale production, and quality control of vaccine production very difficult. As a result of the limitations with the relatively short duration of protection, the lack of cross-protection, and suboptimal safety profile of currently available HS vaccines, the adoption of vaccination protocols in LMICs remains relatively low. Additionally, the logistics of vaccine distribution to small-scale livestock holders in LMICs presents major difficulties to the maintenance of a regular vaccine schedule. Especially considering the rapid onset and progression of HS outbreaks in often challenging landscapes, stringent monitoring and a comprehensive plan for prevention and control would be very beneficial. One component to accomplish this goal is the development of a standardized, highly efficacious vaccine.

Several approaches have been pursued to develop novel antigens as vaccines against bovine strains of *P. multocida* in general and HS in particular. Most of these studies target proteins that are suspected virulence factors and are surface exposed [reviewed by Mostaan et al. (14)]. Previously, outer membrane vesicles from BRD strains of *P. multocida* were isolated and used for immunization of mice, and OmpA (adhesin), OmpH (porin), and P6 were identified as the main immunogenic proteins (15). Vaccination of ducks with recombinant OmpH was protective against intra-nasal challenge in a duck cholera model (16). Ongoing work with OmpH in an HS-specific context has recently led to trials in dairy calves (17) and swamp buffalo (18); however, challenge studies were only performed in buffalo; thus, protective efficacy remains unclear in dairy calves. In addition to this, the outer membrane protein PlpE was identified as a conserved and immunodominant vaccine target and was able to induce protection in mice and chicken (19, 20). While BRD-associated strains of *P. multocida* were found to contain *plpE*, none of the HS-associated *P. multocida* genomes evaluated contained this gene, limiting the potential efficacy of this target for bovine associated infections (21). PlpB was identified as a potential vaccine candidate for bovine pasteurellosis due to its high sequence homology between strains of different origin; however, the recombinant protein only provided partial protection against homologous challenge in mice (22). Few antigens have been queried for their efficacy against multiple serogroups of *P. multocida* infection; however, three novel and undescribed outer membrane proteins from bovine strains were identified and shown to elicit partial protection from challenge with serotypes A, B, and F in mice (23). Other candidates considered for recombinant protein vaccines against *P. multocida* in cattle include PtfA, Oma87, HgbA, and TbpA (7). Oma87 has been evaluated by passive transfer of immune rabbit sera that was protective against challenge in a murine model (24); however, no further challenge studies have been pursued with this antigen. TbpA elicited variable protection in a mouse challenge model of *P. multocida*, with no formulation exceeding 50% protection (25). Considering the limited success in developing novel vaccines for use against bovine *P. multocida* infections, work remains to identify a robust target.

Recently, our group has identified a novel surface lipoprotein, termed PmSLP, that is present in nearly all bovine-associated strains of *P. multocida* evaluated to date (21). PmSLP separates into four distinct phylogenetic clusters, termed PmSLP-1 through PmSLP-4, with high conservation within each cluster but low identity between them. PmSLP-1– and PmSLP-2–containing strains are most often associated with BRD infections in cattle, whereas all HS-causing strains contain the *pmSLP-3* gene. While the functional relevance of PmSLP variants remains to be investigated, the consistent retention of PmSLP in the genomes of bovine-associated strains of *P. multocida* likely indicates that this protein plays an important role in survival and/or pathogenesis in cattle. We previously demonstrated that PmSLP-1 and PmSLP-3 were both highly immunogenic in mice and cattle. Vaccine formulations containing either PmSLP-1 or PmSLP-3 were able to elicit homologous protection in a murine *P. multocida* challenge model, but neither protein elicited cross-protection against strains harboring the other PmSLP. A bivalent vaccine containing both PmSLP-1 and PmSLP-3 extended protection against strains containing either PmSLP variant. In a direct cattle challenge study, animals that were vaccinated twice with PmSLP-3 were protected against challenge with a serogroup B strain of *P. multocida* (21). These previous studies evaluated PmSLP formulated with two commercial adjuvants, aluminum hydroxide gel and Montanide Gel 02 with Poly(I:C), and utilized multiple immunizations with a high amount of protein per dose. Therefore, considerable work remained to identify a final PmSLP-3 vaccine formulation that would be highly efficacious and accessible.

Here, we further evaluate PmSLP-3–based vaccines for their utility as HS vaccines with special consideration for conditions in LMICs. The experimental design was driven by the need to optimize the vaccine formulation to elicit robust, long-lasting protection, provide a high safety profile, and allow use with low effort by veterinarians and livestock owners in LMICs. We optimize the PmSLP-3–based vaccine formulations by evaluating how different adjuvants alter the potential immunogenicity and protective efficacy, and we further determine the minimal antigen concentration and the number of doses required for protection. Duration of protection was evaluated after a single dose of vaccine, where cattle were challenged after 7 months and we demonstrate protection against both serogroups B and E strains of *P. multocida.* Together, these data indicate that a PmSLP vaccine is effective for use against both serogroups of *P. multocida* known to cause HS.

## Materials and methods

### Protein production

Plasmids containing *pmSLP* constructs were transformed into *E. coli* T7 SHuffle, and transformants were selected on Luria-Bertani (LB) agar with ampicillin (100 µg/mL). Multiple colonies were used to inoculate starter cultures in 20 mL of LB with ampicillin (100 µg/mL) and grown at 37°C with shaking for 16 h. Proteins were produced either as cytoplasmic preparations or by protein refolding. No differences in protective efficacy have been noted in mice between the two preparation methods.

For cytoplasmic proteins, overnight cultures were used to inoculate 2 L of LB, and the larger culture was grown at 37°C while shaking for approximately 3 h or until optical density 600nm of 0.5 was reached. Protein expression was induced with the addition of isopropyl β-d-1-thiogalactopyranoside to a final concentration of 0.5 mM, and cells continued to grow overnight with shaking at 20°C. Cells were pelleted at 6,000 *x g* and resuspended in 40 mL of lysis buffer [50 mM Tris-HCl (pH 8.0) and 300 mM NaCl] with 10 mM imidazole (pH 8.0), 1 mM phenylmethylsulfonyl fluoride (PMSF), 1 mM benzamidine, lysozyme (1 mg/mL), and DNaseI (0.03 mg/mL). Cells were lysed by sonication (Branson) for 10 min on ice and centrifuged at 35,000 *x g* for 45 min to remove cell debris. The supernatant was filtered through a 0.45-µm filter and incubated at 4°C for 1 h with 0.5 mL of HisPur Ni-NTA resin (Thermo Fisher Scientific). Beads were pelleted for 5 min at 700 *x g*, loaded onto a gravity column (Econo-Pac Bio Rad), and washed with 100 mL of cold wash buffer [lysis buffer with 20 mM imidazole (pH 8.0)]. Protein was eluted in 10 mL of cold elution buffer [lysis buffer with 400 mM imidazole (pH 8.0)] and incubated with 2 U of bovine thrombin (cat. T4648, Sigma-Aldrich) to remove the poly-histidine purification tag in dialysis against 1 L of 20 mM Tris-HCl (pH 8.0) and 100 mM NaCl at 4°C overnight. Dialyzed sample was incubated with 100 µL of HisPur Ni-NTA resin (Thermo Fisher Scientific) and 100 µL of p-aminobenzamidine-agarose (Sigma-Aldrich) for 1 h with rotation at 4°C. Cleaved proteins were concentrated to 20 mg/mL by 10K molecular weight cut-off (MWCO) concentrator (Thermo Fisher Scientific), centrifuged at 10,000 *x g* for 10 min at 4°C to remove aggregates, and purified by size exclusion chromatography (Superdex 200 10/300 GL, GE Healthcare).

For refolded protein antigens, the overnight cultures were used to inoculate 1.5 L of ZY auto-induction medium containing ampicillin (50 µg/mL), and the larger culture was grown overnight at 37°C. Cells were harvested via centrifugation at 6,000 *x g*, and pellet was resuspended in 50 mL of lysis buffer [50 mM Tris-HCl (pH 8.0), 300 mM NaCl, and 10 mM imidazole (pH 8.0)], with 1 mM PMSF, 1 mM benzamidine, lysozyme (1 mg/mL), and DNaseI (0.03 mg/mL). Cells were lysed by sonication (Branson) for 10 min on ice and centrifuged at 35,000 *x g* for 45 min. The layer of cell debris covering the inclusion body (IB) pellet was washed away with ddH_2_O. The IB pellet was then resuspended in at least 30 mL of denaturing buffer [50 mM Tris-HCl (pH 8.0), 200 mM NaCl, and 6 M Urea]. Once the pellet was completely solubilized, sample was filtered through a 0.45-µm filter and added to the refolding buffer [50 mM Tris (pH 8.0), 300 mM NaCl, and 400 mM L-Arginine] in a dropwise manner with constant stirring. For every 20 mL of supernatant, 100 mL of refolding buffer was used. The resulting solution was filtered through a 0.22-µm filter and loaded onto a 5-mL HisTrap Excel column (GE Healthcare) using an Amersham peristaltic pump at a flow rate of 1 mL/min. The column was then loaded on to an AKTA Pure system, washed in wash buffer [50 mM Tris-HCl (pH 8.0) and 300 mM NaCl] until the UV signal reached baseline. The protein was then eluted in elution buffer [50 mM Tris-HCl (pH 8.0), 300 mM NaCl, and 400 mM Imidazole (pH 8.0)]. Selected fractions were pooled and dialyzed against 1 L of 20 mM Tris-HCl (pH 8.0) and 100 mM NaCl at 4°C overnight. Dialyzed sample was concentrated to 20 mg/mL by 10K MWCO concentrator (Thermo Fisher Scientific), centrifuged at 10,000 *x g* for 10 min at 4°C to remove aggregates, and purified by size exclusion chromatography (Superdex 200 10/300 GL, GE Healthcare).

For antigen studies, PmSLP protein was further purified on a strong anion exchange chromatography column (MonoQ 5/50 GL, GE Healthcare) to remove endotoxins. Cytoplasmic protein preparations were used for mouse immunization and enzyme-linked immunosorbent assay (ELISA) studies, cattle trials 1 and 2, and cattle plasma ELISAs, whereas refolded protein was used for cattle trials 3 through 6 and cattle serum ELISAs.

### Mouse immunization study

Five different formulations of PmSLP-1 vaccine were evaluated in C57Bl/6 mice. A mix of male and female mice (n = 5 male, n = 4 female, 5–6 weeks of age, Charles River) were purchased and allowed to acclimate in the University of Toronto mouse facility. After acclimation, mice received PmSLP-1 vaccine [20 µg of PmSLP-1 diluted in sterile phosphate-buffered saline (PBS) and formulated with the appropriate adjuvant] via sub-cutaneous (s.c.) injection on day 0 and day 21. Serum samples were obtained after each dose, as well as multiple times up to 26 weeks after the second immunization. Adjuvants used were alum (100 µg per dose, Alhydrogel, InvivoGen, San Diego, CA), Emulsigen-D (20% v/v, oil-in-water based, MVP, Teaneck, NJ), Montanide Gel 02 (20% v/v, polymer-based, Seppic, France), Montanide Gel 02 with Poly I:C [20% v/v Montanide Gel 02, 3 µg Poly(I:C) per dose, InvivoGen, San Diego, CA], and Montanide ISA 61 (60% v/v, water-in oil based, Seppic, France). Montanide ISA 61 requires using high shear force to create a stable emulsion, which was obtained through homogenization [three 10-s iterations at a speed of approximately 14,500 rpm (IKA T10 Basic S1 homogenizer)].

Mouse studies were performed at the University of Toronto. Mice were handled humanely under animal use protocol 20011319, approved by the animal care committee at the University of Toronto. Mice were maintained in specific pathogen–free conditions and were provided water and rodent chow *ad libitum.*

### Mouse serum ELISA

Mouse serum samples were evaluated by protein ELISA as described previously (21). Briefly, 20 µL of serum was added in two-fold dilutions in 1% bovine serum albumin (BSA) starting at a dilution of 1:250 up to 1:32,000 for subclass evaluation and 1:8,000 and up to 1:1,024,000 for total immunoglobulin G (IgG) and duration analysis to PmSLP-coated plates that were already washed with PBS with 0.05% Tween-20 (PBST) and blocked with 5% BSA and then incubated at 4°C overnight. Plates were washed and 20 µl of either goat anti-mouse IgG H&L (HRP) (cat. ab6789, Abcam; dilution 1:10,000); alkaline phosphatase AffiniPure™ goat anti-mouse IgG, Fcγ subclass 1 specific (cat. 115-005-205, Jackson ImmunoResearch Laboratories, Inc., Canada; dilution 1:5,000); alkaline phosphatase AffiniPure™ goat anti-mouse IgG, Fcγ subclass 2b specific (cat. 115-005-207, Jackson ImmunoResearch Laboratories, Inc., Canada, dilution 1:5,000); or alkaline phosphatase AffiniPure™ goat anti-mouse IgG, Fcγ subclass 2c specific (cat. 115-005-208, Jackson ImmunoResearch Laboratories, Inc., Canada, dilution 1:5,000) was added per well and incubated for 2 h at room temperature. Plates were washed and developed with KPL SureBlue™ TMB 1-Component Microwell Peroxidase Substrate for HRP-labeled antibodies (cat. 5420-0028, SeraCare, Canada), and the reaction was stopped with 2N sulfuric acid or with KPL BluePhos Microwell Phosphatase Substrate System for AP-labeled antibodies (cat 5120-0059, SeraCare, Canada). Absorbance was read at 450 nm/570 nm for HRP-labeled plates or 620 nm for AP-labeled plates. Endpoint titres were determined as the last dilution where the absorbance was at minimum three standard deviations higher than the mean background signal. Serum samples were tested in duplicate.

### Cattle immunization studies

All cattle studies are summarized in **Table 1**. Trials 1 and 2 were performed at the University of Calgary’s Veterinary Services Research Station under approved animal use protocol AC20-0007. Calves were sourced from a high-health commercial dairy operation and tested free (based upon antibody titres) from BVDV, Bovine Leukosis and Neospora. Before arrival at the research station, animals had been immunized with BoviShield OneShot Gold Viral/*M. haemolytica* (Zoetis, US) and a multivalent Clostridia vaccine at 6 and 12 weeks of age, as well as against *Moraxella bovis* at 12 weeks of age. The animals had not been immunized for BRD associated with *P. multocida*. Animals were housed in outdoor pens for the first 6 months and fed hay *ad libitum* and non-medicated starter feed once per day. At approximately 12 months of age, animals were integrated into a larger herd on pasture for the duration of immunity study.

**Table 1:** Cattle trials performed within this program.

Trial	Location	Animals	Vaccinations	Sampling and challenge
1	University of Calgary, Canada	6-month-old female Holstein cattle	• Two doses s.c. (day 0 and day 21) • 200 µg of PmSLP-3 formulated with AlOH, Montanide Gel 02 + Poly(I:C), or Montanide ISA 61 (n = 5 for all groups)	• Blood samples taken at day 0, day 21, day 56, and monthly from 3 months to 12 months. Final sample taken at 36 months. Plasma stored at −20°C until analysis. • No challenge
2	University of Calgary, Canada	6-month-old female Holstein cattle	• One or two doses i.m. (day 0, or day 0 and day 21) • 50-µg or 100-µg doses of PmSLP-3 formulated with Montanide ISA 61 (n = 3 for all groups)	• Blood samples taken at day 0, day 21, day 56, and at 12 months and 18 months after immunization. Plasma stored at −20°C until analysis. • No challenge
3	National Veterinary Institute, Ethiopia	1- to 2-year-old male Zebu cattle	• Two doses s.c. (day 0 and day 21) • 200 µg of PmSLP-3 formulated with either Alk(SO_4_)_2_, AlOH, or Montanide ISA 61; serogroup B Bacterin [Alk(SO_4_)_2_ adjuvanted]; adjuvant only [Alk(SO_4_)_2_] (n = 8 for all groups)	• Challenge at 2 weeks after booster vaccination • S.c. infection of 5.3 × 10^5^ CFU of serogroup B strain
4	National Veterinary Institute, Ethiopia	1- to 2-year-old male Zebu cattle	• One or two doses s.c. (day 0 and day 21 for two dose cohort, for single dose cohort, vaccine was given on day 21 and adjuvant only was given on day 0 to maintain study blinding) • 50 µg or 100 µg of PmSLP-3 formulated with Montanide ISA 61 or Montanide ISA 61 only (n = 10 for all groups)	• Challenge at 3 weeks after booster vaccination • S.c. infection of 5.3 × 10^5^ CFU of serogroup B strain
5	National Veterinary Institute, Ethiopia	1- to 2-year-old male Zebu cattle	• Single dose s.c. for PmSLP-3, two doses for serogroup B Bacterin • 100 µg of PmSLP-3 + Montanide ISA 61 (n = 10); local Bacterin (n = 10); non-vaccinated, naïve calves (n = 8)	• Challenge 31 weeks after vaccination • S.c. infection of 4.5 × 10^5^ CFU of serogroup B strain
6	National Veterinary Institute, Ethiopia	1- to 2-year-old male Zebu cattle	• Single dose s.c. for PmSLP-3, two doses for serogroup B Bacterin • 100 µg of PmSLP-3 + Montanide ISA 61; serogroup B Bacterin; non-vaccinated, naïve calves (n = 10 for all groups)	• Challenge at 5 weeks after vaccination • S.c. infection of 6.5 × 10^5^ CFU of serogroup E strain

s.c., sub-cutaneous; i.m., intra-muscular; CFU, colony-forming units.

#### Trial 1: adjuvant selection

Groups of 6-month-old female Holstein calves were immunized twice with 200 µg of PmSLP-3 formulated with different adjuvants. Groups of five calves each were randomly assigned to adjuvant groups and received 1 mL of vaccine s.c. on day 0 and day 21. The adjuvants used were an aluminium hydroxide gel (Alhydrogel 2%, InvivoGen, San Diego, CA), a proprietary polymer adjuvant (20% v/v, Montanide Gel 02, Seppic, France) formulated with Poly I:C (InvivoGen, San Diego, CA), and a proprietary water-in-oil adjuvant (60% v/v, Montanide ISA 61, Seppic, France). All adjuvants were used according to the manufacturer’s instructions. Montanide ISA 61 required high-shear emulsification, which was achieved by mixing for 10 min on ice with a handheld homogenizer at the highest setting (TH Emulsifier with Flat bottom [fine] probe, Omni International, Kennesaw, GA, US). Ethylenediaminetetraacetic acid (EDTA)-blood and mucosal swabs from the nasal cavity were collected on day 0, day 21, and day 56, then once per month up to 12 months after immunization, and one final collection at 36 months after immunization. Nasopharyngeal swabs were collected with sterile double-guarded swabs (double-guarded equine uterus swabs, Reproduction Resources, Walworth, WI, USA).

#### Trial 2: dose study

Six-month-old female Holstein calves, sourced from the same herd and subjected to the same health testing and vaccination program as trial 1, were randomly assigned to four immunization groups. Three animals each were immunized intra-muscularly (i.m.) with 1 mL of PmSLP-3 vaccine adjuvanted with Montanide ISA 61 (60% v/v Seppic, France). The protein dosage groups were 50 µg, one immunization (day 0); 50 µg, two immunizations (day 0 and day 21); 100 µg, one immunization (day 0); and 100 µg, two immunizations (day 0 and day 21). EDTA blood was collected for serology after each immunization, as well as at 12 months and 18 months after immunization.

### Cattle plasma ELISAs

Protein (100 ng per well) was immobilized on 96-well microtitre plates (Nunc MaxiSorp) overnight at 4°C and washed three times with PBST, then plates were blocked with non-animal protein blocking buffer (NAP blocker, g-biosciences, St. Louis. MO). EDTA plasma was diluted in two-fold serial dilutions from 1:10,000 to 1:1,280,000 for animals vaccinated with Montanide ISA 61–formulated antigen and from 1:100 to 1:12,800 for animals vaccinated with other adjuvant formulations and 100 µL were added per well. Plasma was incubated for 2 h at room temperature. Plates were washed and 100 µl of either sheep anti-bovine IgG : HRP secondary antibody (cat. AAI23P, BioRad, Canada, dilution 1:10,000), sheep anti-bovine IgG1:HRP (cat. AAI21P, BioRad, Canada, dilution 1:10,000), or sheep anti-bovine IgG2:HRP (cat. AAI22P, BioRad, Canada, dilution 1:10,000) was incubated for 2 h at room temperature. Plates were washed and developed with TMB substrate (Thermo Scientific 1-step TMB ELISA substrate). To stop the reaction, 100 µL of 1N sulfuric acid was added, and absorbance was read immediately at 450 nm. Samples were run parallel with the corresponding null sera, and endpoint titres were determined as the last dilution where the absorbance was above background (three standard deviations above null sera). Samples were run in triplicates on three separate plates for a total of nine data points per sample.

### Cattle challenge studies

Cattle challenge studies were performed at the National Veterinary Institute (NVI) in Debre Zeyit, Ethiopia, and are summarized in **Table 1** (studies 3 to 6). All cattle utilized were sourced from local commercial livestock markets and were apparently healthy and had no history of vaccination for HS. All calves were sero-negative for *P. multocida* by indirect hemagglutination assay. Calves were dewormed using broad spectrum anthelmintics and acclimated to the facility for a minimum of 2 weeks prior to the start of any immunization process. Throughout the studies, concentrate supplement (wheat bran) and fresh water were provided *ad libitum.*

Vaccines for cattle trial 3 were formulated at the University of Toronto. For PmSLP-3 vaccines formulated with either aluminum hydroxide gel or aluminum potassium sulfate, PmSLP-3 was lyophilized in sterile vials. Aluminum hydroxide gel was diluted in PBS to a final concentration of 0.15% Alhydrogel, whereas aluminum potassium sulfate was diluted in PBS to a final concentration of 1% Alk(SO_4_)_2_. Both alum diluents were shipped in separate vials and reconstituted with the lyophilized PmSLP-3 immediately prior to vaccination by basic mixing. As Montanide ISA 61 requires high shear to achieve a stable emulsion, vaccines were formulated at the University of Toronto by mixing PmSLP-3 diluted appropriately in PBS with 60% v/v Montanide ISA 61. Vaccine was then homogenized (IKA T10 Basic S1 homogenizer) for 3 min at speed 2 (approximately 10,000 rpm) followed twice homogenizing for 3 min at the maximum speed (approximately 30,000 rpm). Stable emulsions were confirmed by “drop floating” the formulation on water. Vaccines for trial 3 were shipped unblinded due to the visual differences in formulations and were stored at 4°C until use. Vaccines for cattle studies 4, 5, and 6 were formulated in a single large batch at the University of Toronto. Montanide ISA 61 (60% v/v) was mixed with PmSLP-3 diluted in PBS to the final concentration specified in each trial below and mixed via pipetting up and down with a 10-mL serological pipette. Vaccines were then homogenized as above, and both doses of vaccine and the adjuvant only Montanide ISA 61 formulation were shipped blinded to NVI and stored at 4°C until use. All calves were monitored for local reactions after immunization. For all challenge studies, cattle were monitored for 7 to 10 days after infection. The lung, liver, and spleen were tested for viable *P. multocida* for all cattle that did not survive the infection, and blood and tissues were tested for PCR-based detection. For all animals that survived, blood samples were taken at the end of the clinical monitoring to test for *P. multocida* via molecular detection. One per group of surviving animals was sent for *post-mortem* analysis for blood, lung, liver, and spleen detection of *P. multocida* by both culture and molecular detection.

#### Trial 3: adjuvant protection study

Forty Zebu cattle were randomly assigned to five groups (n = 8 cattle per group): PmSLP-3 formulated with AlOH (1 mg of aluminum per dose, Alhydrogel, InvivoGen, San Diego, CA), PmSLP-3 formulated with Montanide ISA 61 (60% v/v, Seppic, France), PmSLP-3 formulated with Alk(SO_4_)_2_ (1% w/v), and the local serogroup B Bacterin vaccine [adjuvanted with 1% w/v Alk(SO_4_)_2_, or Alk(SO_4_)_2_ only (adjuvant only)]. PmSLP-3–containing vaccines included 200 µg of protein, and all vaccines were prepared as 2 mL per dose in sterile PBS. Animals were immunized twice s.c. on day 0 and day 21. Blood was collected prior to the first dose and after each immunization. The animals were challenged s.c. with a 6-h culture of 5.3 × 10^5^ colony-forming units (CFU) of a local serogroup B HS strain of *P. multocida* 2 weeks after the second immunization.

#### Trial 4: required dose study

Fifty Zebu cattle were randomly assigned to five groups (n = 10 per group). Vaccines were composed of 2 mL of PmSLP-3 vaccine formulated with Montanide ISA 61 (60% v/v, Seppic, France) and were delivered s.c. Two groups received a single vaccine dose (adjuvant only on day 0; 50 µg or 100 µg of PmSLP-3–containing vaccine on day 21), whereas two groups received two doses (50 µg or 100 µg, day 0 and day 21). The fifth group received two doses of adjuvant only. Cattle were challenged with a 6-h culture of 5.3 × 10^5^ CFU of a local serogroup B HS strain of *P. multocida* 3 weeks after the second immunization.

#### Trial 5: duration of protection study

Twenty-eight Zebu cattle were randomly assigned to PmSLP-3 vaccine group (n = 10), local Bacterin group (n = 10), or control (naïve, n = 8). Cattle received a single dose (day 0) of 2 mL of vaccine s.c. (100 µg of PmSLP-3 vaccine formulated with 60% v/v Montanide ISA 61) or two doses (day 0 and day 21) of the serogroup B bacterin [adjuvanted with 1% w/v Alk(SO_4_)_2_]. Serum samples were collected prior to vaccination and at five time points after immunization up until challenge. At 31 weeks after immunization, cattle were challenged with a 6-h culture of 4.5 × 10^5^ CFU of a local serogroup B HS strain of *P. multocida.*

#### Trial 6: serogroup E protection study

Thirty Zebu cattle were randomly assigned to PmSLP-3 vaccine group (n = 10), local Bacterin group (n = 10), or control (naïve, n = 10). Cattle received a single dose (day 0) of 2 mL of vaccine s.c. (100 µg of PmSLP-3 vaccine formulated with 60% v/v Montanide ISA 61) or two doses (day 0 and day 21) of the serogroup B bacterin [adjuvanted with 1% w/v Alk(SO_4_)_2_]. Serum samples were collected prior to vaccination and at 3 and 5 weeks after immunization. At 5 weeks after immunization, cattle were challenged with a 6-h culture of 6.5 × 10^5^ CFU of a local serogroup E HS strain of *P. multocida.*

### Cattle serum ELISAs

ELISA kits were assembled at the University of Toronto and sent to NVI, Ethiopia, for cattle serum serological analysis, as previously described (21).

### Statistical analysis

Statistical evaluations were performed in Prism 10. For all challenge studies, Log-rank (Mantel–Cox) tests were performed to compare the survival curves of each individual immunized group versus the adjuvant control group. Antibody titres were evaluated using one- or two-way ANOVA, where appropriate, with repeated measures and *post-hoc* analysis as described in figure legends. *p < 0.05, **p < 0.01, ***p < 0.001, and ****p < 0.0001; ns, not significant.

## Results

### Selection of adjuvant for inclusion in a PmSLP vaccine formulation

Our previous challenge studies performed in cattle (21) demonstrated that both Montanide Gel 02 + Poly(I:C) and aluminum hydroxide gel adjuvanted PmSLP-3 vaccines elicited protection in Zebu cattle; however, this protection was not as robust as protection seen in cattle that received the locally produced serogroup B bacterin, where all cattle survived challenge with *P. multocida*. Therefore, as a first step to improving PmSLP-mediated protection, we sought to evaluate different vaccine formulations for their ability to elicit humoral antibody responses in mice. Toward determining what adjuvant would be most effective when formulated with PmSLP, we evaluated the immunogenicity of five different PmSLP-1 vaccine preparations in C57Bl/6 mice. These were formulated with a gel-based polymeric adjuvant (Montanide Gel 02) with or without the TLR-3 agonist Poly(I:C), the oil-in-water-based Emulsigen-D, the water-in-oil-based Montanide ISA 61, or aluminum hydroxide gel (alum). PmSLP-1 was used here as initial studies evaluated protection against both BRD- and HS-causing strains of *P. multocida*, and no substantial differences have been noted between antibody responses to PmSLP-1 versus PmSLP-3.

**Figure 1:**
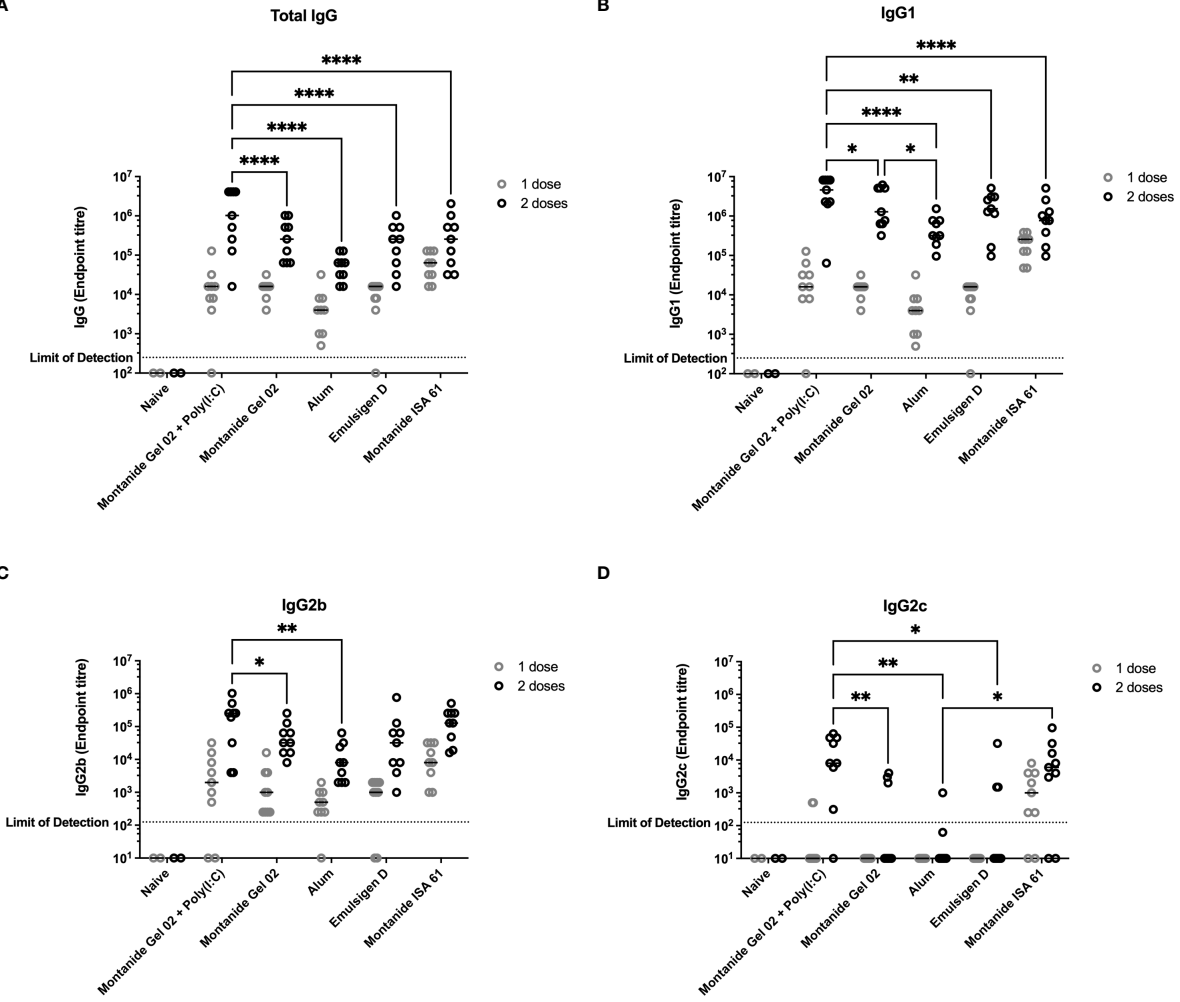
Immunogenicity and antibody subclasses present in sera from C57Bl/6 mice immunized with different PmSLP-1 formulations. Antisera from individual mice (n = 2 mice for naïve and n = 9 mice for each vaccinated groups) were tested in technical duplicate for **(A)** total IgG, **(B)** IgG1, **(C)** IgG2b, and **(D)** IgG2c after one dose (gray circles) and two doses (black circles) of vaccine. Titres were compared with a two-way ANOVA with multiple comparisons with Uncorrected Fisher’s least significant difference (LSD) at each time point. Line represents median. Comparisons to naïve were ignored due to the naïve group being under powered (n = 2) for analysis. Only significant differences are indicated, other comparisons are not significant, * represents p ≤ 0.05, ** represents p ≤ 0.01, and **** represents p ≤ 0.0001.

All mice that received PmSLP-1 vaccines elicited an anti–PmSLP-1 serum IgG response by the second dose (**Figure 1A**), and all mice that received PmSLP-1 vaccines formulated with alum, Montanide Gel 02, and Montanide ISA 61 elicited detectable IgG after only one dose. Interestingly, Montanide Gel 02 + Poly(I:C)–formulated vaccine elicited the highest level of PmSLP-1 specific total IgG (**Figure 1A**) and IgG1 (**Figure 1B**). When considering antibodies more correlated with functional responses such as bactericidal activity, PmSLP-1 formulated with Montanide Gel 02 + Poly(I:C) elicited higher IgG2b compared to formulations containing Montanide Gel 02 alone or alum (**Figure 1C**) and higher IgG2c compared to formulations containing Montanide Gel 02, alum, or Emulsigen-D (**Figure 1D**). PmSLP-1 formulated with Montanide ISA 61 elicited the highest total IgG after the first dose and elicited higher levels of IgG2c than the formulation containing alum and was not significantly different compared to the formulation containing Montanide Gel 02 + Poly(I:C). PmSLP-1 vaccine containing Montanide ISA 61 elicited the highest level of IgG and all IgG subclasses tested after a single dose; however, this was not statistically significant.

**Figure 2:**
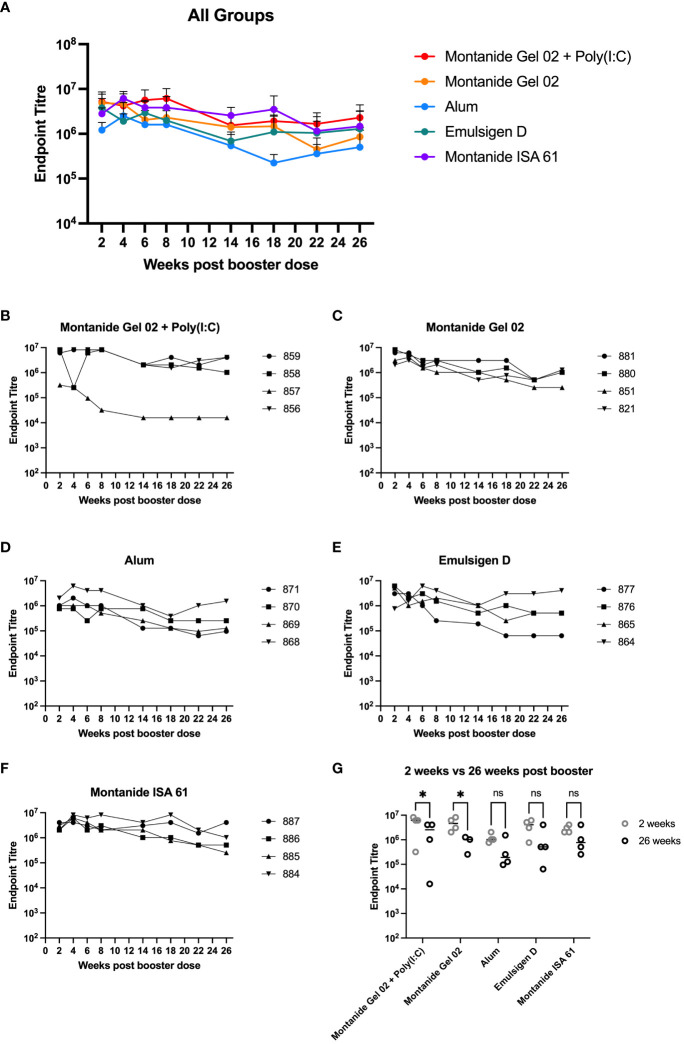
Duration of immunity in mice. Persistence of vaccine elicited antibodies for 26 weeks after booster for all groups **(A)** and separated by individual mice for each formulation including PmSLP-1 formulated with Montanide Gel 02 + Poly(I:C) **(B)**, Montanide Gel 02 **(C)**, Alum **(D)**, Emulsigen-D **(E)**, and Montanide ISA 61 **(F)**. Titres at 26 weeks after booster are compared to titres at 2 weeks after booster **(G)**. Titres were compared with a two-way ANOVA with multiple comparisons with uncorrected Fisher’s LSD comparing titres from each formulation at 2 weeks and 26 weeks. Line represents median for panel **(G)**. * represents p < 0.05, ns represents non-significant difference.

A subset (n = 4 per formulation) of the mice that received different PmSLP-1 formulations were maintained for 26 weeks after receiving the booster dose. Sera from these mice were sampled at eight time points from 2 to 26 weeks after booster. These samples were evaluated for the maintenance of anti–PmSLP-1 IgG (**Figures 2A–F**). In general, reactivity remained high at all time points evaluated for all formulations, with mice maintaining high levels of serum IgG throughout the duration evaluated. One mouse that received PmSLP-1 formulated with Montanide Gel 02 + Poly(I:C) had a substantial drop in immunogenicity compared to the other mice in that group, which maintained high levels of antibodies throughout. PmSLP-1 formulated with Emulsigen-D elicited the most variable animal to animal response while also showing the most variation across each time point. Notably, PmSLP-1 formulated with Montanide ISA 61, Montanide Gel 02, and Montanide Gel 02 + Poly(I:C) elicited high levels of PmSLP-specific antibody that showed no indication of waning over the time span evaluated. When comparing the serum IgG levels of PmSLP-1 antibodies at 26 weeks after booster to the titres at 2 weeks after booster, all titres remained high; however, there was a statistically significant drop for both PmSLP-1 formulated with Montanide Gel 02 alone and formulated with Montanide Gel 02 + Poly(I:C) (**Figure 2G**).

**Figure 3:**
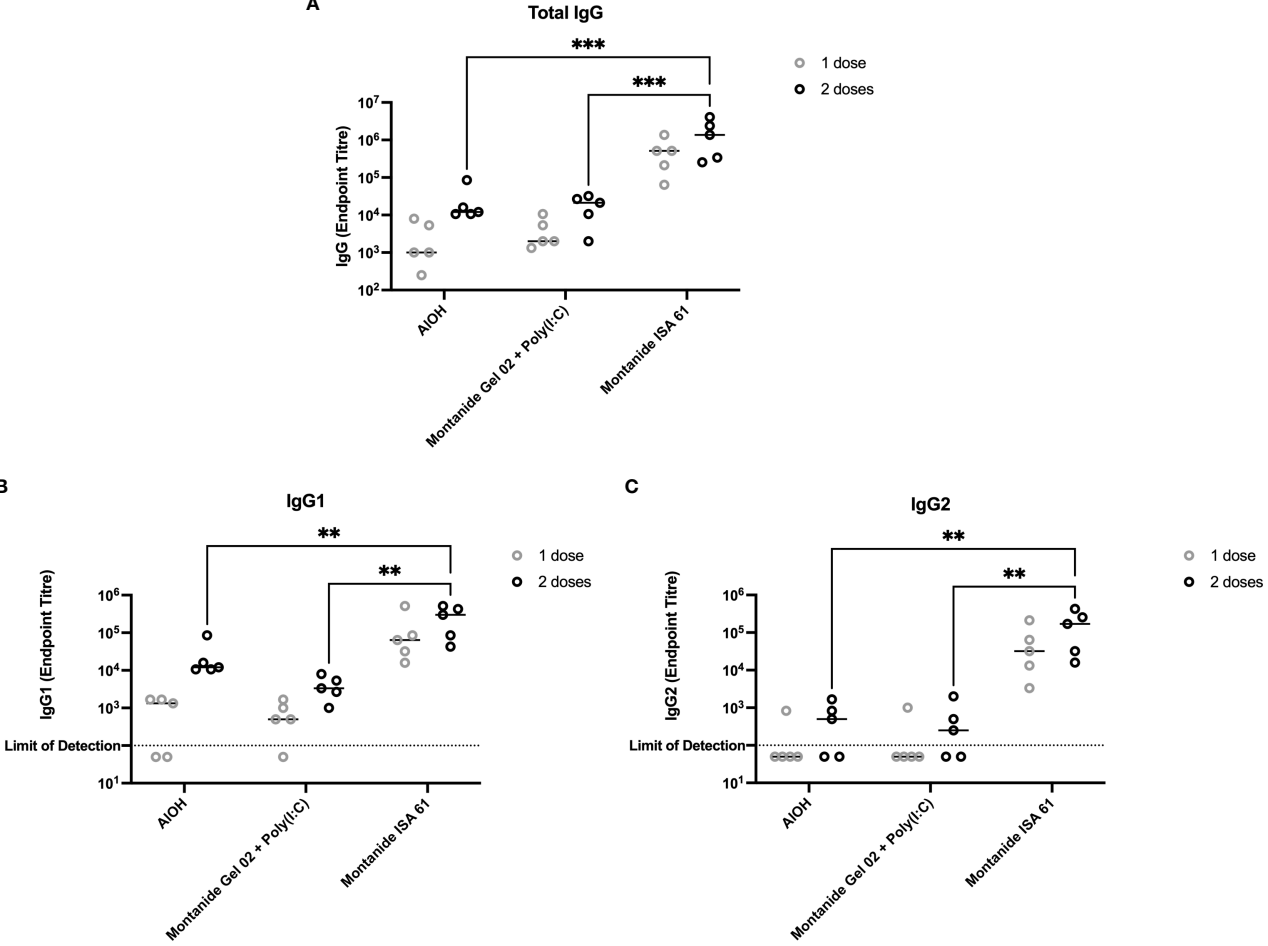
Immunogenicity and antibody subclasses present in plasma from Holstein cattle immunized with different PmSLP-3 adjuvant formulations. Plasma from individual calves was tested by ELISA for PmSLP-3–specific **(A)** IgG, **(B)** IgG1, and **(C)** IgG2. Samples from each calf at each time point were tested in triplicate on three separate plates for a total of nine technical data points per sample, with the line representing the median. Performed as part of cattle trial 1 at the University of Calgary. Titres were compared with a two-way ANOVA with multiple comparisons with uncorrected Fisher’s LSD at each time point. Only significant differences are indicated, other comparisons are not significant, ** represents p ≤ 0.01, and *** represents p ≤ 0.001.

A subset of the adjuvant formulations under evaluation were next tested in 6-month-old Holstein cattle (cattle trial 1). Montanide ISA 61 and Montanide Gel 02 + Poly(I:C) formulations were selected as they were the most promising in mice and elicited higher levels of functional antibody classes (IgG2b and IgG2c) compared to other formulations. Alhydrogel (alum) was selected as aluminum-based adjuvants are widely used, are reasonably low cost, are generally well tolerated, and Alhydrogel is easy to formulate. All formulations were immunogenic (**Figure 3A**); however, PmSLP-3–specific serum IgG was highest in cattle that received vaccine formulated with Montanide ISA 61 (**Figure 3A**), even after a single dose. Additionally, Montanide ISA 61 formulations elicited IgG1, the antibody subclass most able to elicit complement-dependent cytotoxicity (26), and IgG2, the antibody subclass most able to bind neutrophils and monocytes (26), in all cattle (**Figures 3B, C**).

**Figure 4:**
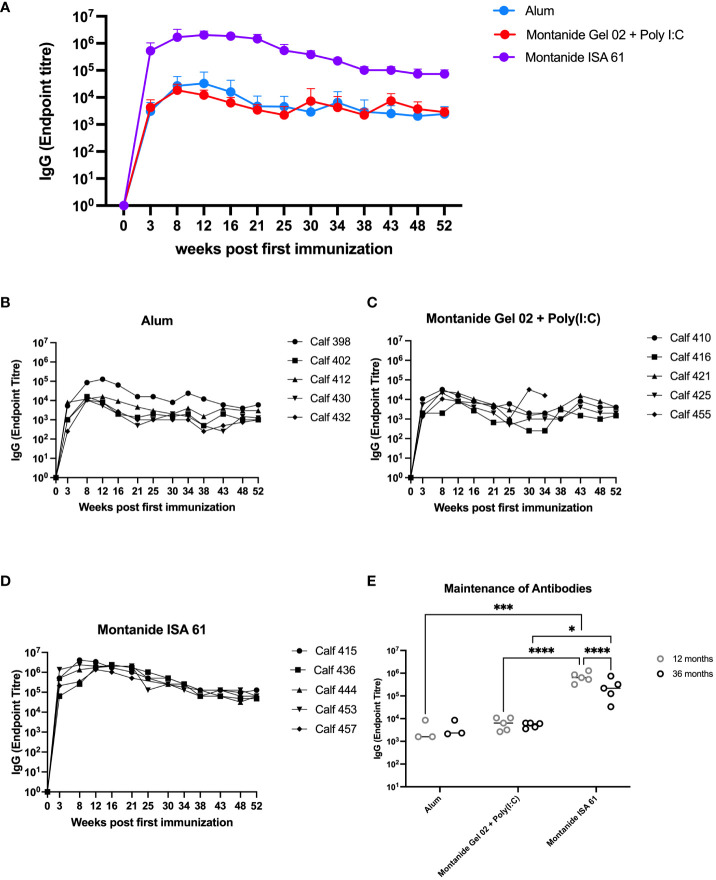
Immunization of cattle with PmSLP-3 antigen for adjuvant selection. Groups of five 6-month-old Holstein calves were immunized with 200 µg of PmSLP-3 formulated with AlOH, Montanide Gel 02 + Poly(I:C), or Montanide ISA 61 adjuvant according to the manufacturer’s instructions. Blood samples were collected for 12 months, and anti–PmSLP-3 plasma IgG was measured by ELISA. **(A)** Average of all animals per group shown, as well as titres of each calf from animals that received PmSLP-3 vaccine formulated with **(B)** aluminum hydroxide gel (alum), **(C)** Montanide Gel 02 + Poly(I:C), or **(D)** Montanide ISA 61. Plasma samples were also obtained at 3 years (36 months) after booster and compared to the titres obtained at 12 months after booster **(E)**. Performed as part of cattle trial 1 at the University of Calgary. Titres were compared with a two-way ANOVA with multiple comparisons with uncorrected Fisher’s LSD comparison. Line represents median for panel **(E)**. Only significant differences are indicated, other comparisons are not significant, * represents p ≤ 0.05, *** represents p ≤ 0.001, and **** represents p ≤ 0.0001.

Based on these results, we extended the evaluation on the effect of adjuvant on immunogenicity and duration of immunity in cattle. Plasma samples were collected approximately monthly over the course of 12 months from cattle that had received PmSLP-3 formulated with alum, Montanide Gel 02 + Poly(I:C), or Montanide ISA 61, as well as a final sample that was taken at 36 months after booster. Animals that received PmSLP-3 formulated with Montanide ISA 61 had approximately 2 log higher titres of anti–PmSLP-3 plasma IgG compared to animals that received formulations containing either alum or Montanide Gel 02 + Poly(I:C), and these antibodies persisted to a higher level over the 12 months after immunization (**Figures 4A–D**). Additionally, when plasma samples were obtained at 3 years after booster, antibodies were maintained in all groups, with cattle that had received PmSLP-3 vaccine formulated with Montanide ISA 61 still maintaining the highest antibody titres (**Figure 4E**).

**Figure 5:**
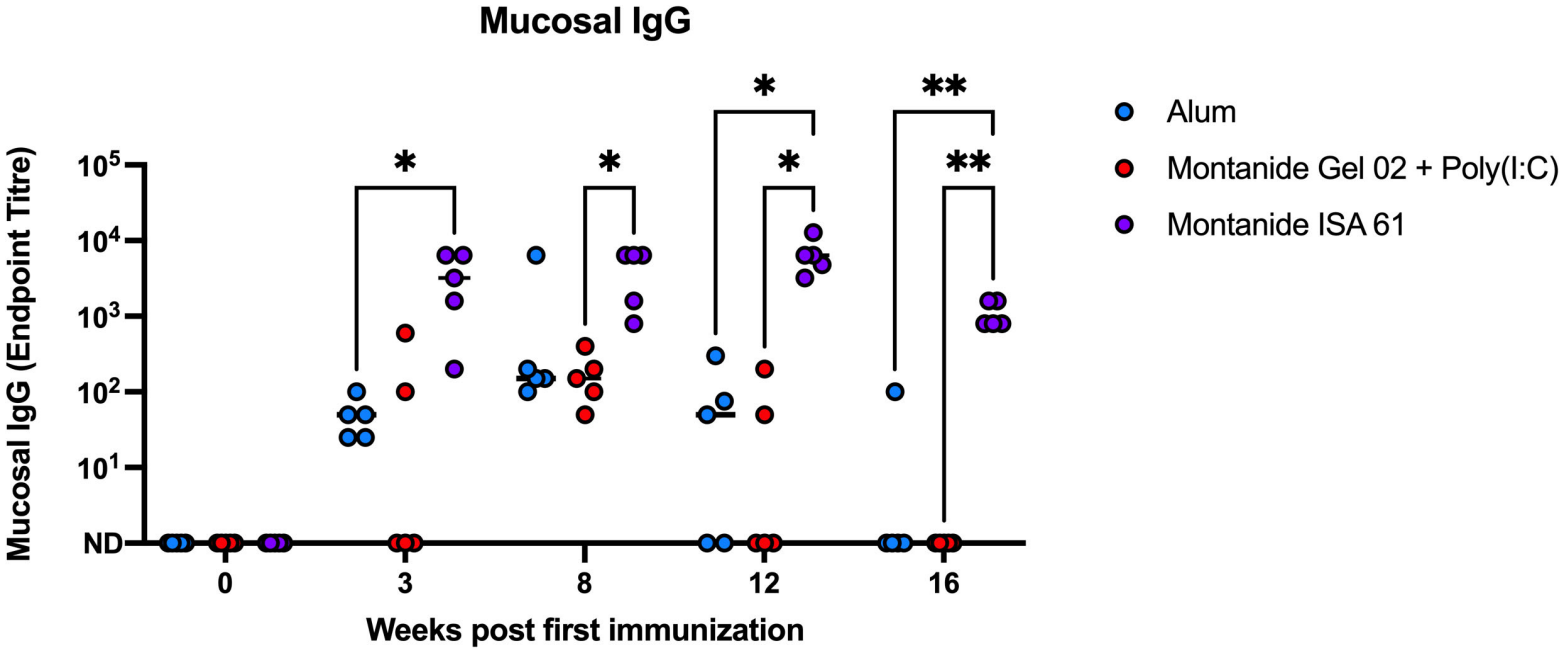
Mucosal IgG endpoint titres for each Holstein calf immunized s.c. with PmSLP-3 formulated with aluminum hydroxide gel (blue), Montanide Gel 02 + Poly(I:C) (red), or Montanide ISA 61 (purple). Data are shown at weeks after first immunization, with doses given at day 0 and day 21. Performed as part of cattle trial 1 at the University of Calgary. Titres at each time point were compared with a repeated-measures two-way ANOVA with multiple comparisons with Tukey’s multiple comparisons test. Line represents median. Only significant differences are indicated, other comparisons are not significant, * represents p ≤ 0.05, and ** represents p ≤ 0.01.

Because *P. multocida* inhabits the bovine upper respiratory tract and is transmitted via oronasal route, mucosal immunity may reduce carriage and transmission during HS outbreaks. Mucosal swabs were collected for 4 months after immunization from the nasal cavity of immunized cattle and tested by ELISA for PmSLP-3–specific mucosal IgG. Consistent with IgG titres in plasma, immunization with Montanide ISA 61–formulated PmSLP-3 vaccine induced mucosal IgG titres that were approximately 2 log higher compared to other formulations, and these antibodies were maintained for a longer duration after immunization (**Figure 5**). In comparison, calves that received vaccine formulated with alum or Montanide Gel 02 + Poly(I:C) saw mucosal titres peak at 5 weeks after booster dose (8 weeks after first immunization) and became undetectable in most animals between 3 months and 4 months after immunization. PmSLP-3–specific mucosal IgA was not detected in these animals after immunization (data not shown).

To evaluate the impact on protection by different adjuvant formulations, a challenge study (cattle trial 3) was performed at the National Veterinary Institute (NVI) in Debre Zeyit, Ethiopia. One- to 2-year-old Zebu cattle (n = 8 per group) were immunized s.c. with 200 µg of PmSLP-3 antigen formulated with aluminum hydroxide gel (alum, AlOH), aluminum potassium sulfate [AlK(SO_4_)_2_], or Montanide ISA 61. Montanide ISA 61 was selected as it was the most promising based on serology data in Holstein cattle and elicited long-lasting titres (shown above), whereas aluminum hydroxide gel was chosen due to ease of use and its inclusion in the previous PmSLP-3 cattle challenge studies (21). Aluminium potassium sulfate was included as an adjuvant in this study due to its cost effectiveness and due to its use in the formulation of *P. multocida* bacterin vaccines currently produced by NVI. Cattle were monitored for vaccination site reactogenicity, revealing that two of the eight cattle that received the local bacterin or adjuvant only [AlK(SO_4_)_2_] had swelling at the injection site, three of the eight cattle that received PmSLP-3 formulated with either alum-based adjuvant had local swelling, and all (eight of the eight) cattle that received PmSLP-3 formulated with Montanide ISA 61 had local reactions. No severe reactions were observed with these formulations and, other than local swelling, the vaccines were well tolerated.

**Figure 6:**
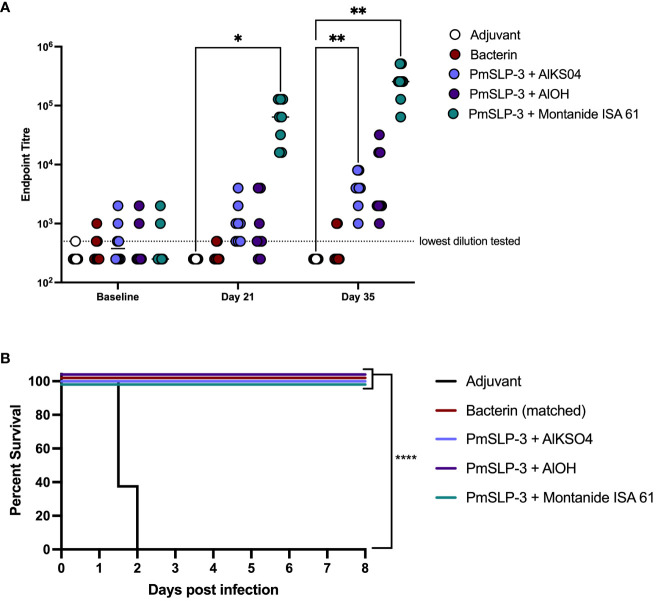
Evaluation of different adjuvants on immunogenicity and protection by PmSLP-3 vaccine formulations. **(A)** Immunogenicity of sera from Zebu cattle that received either aluminum potassium sulfate only (Adjuvant only, “AlkSO4,” white), the local Bacterin (red), or PmSLP-3 formulated with aluminum potassium sulfate (light purple), aluminum hydroxide gel (AlOH, dark purple), or Montanide ISA 61 (teal) against purified PmSLP-3 protein. Serum was evaluated at baseline (day 0), after one dose of vaccine (day 21), or after two doses of vaccine (day 35). Each dot represents the mean of technical triplicates with line representing median. **(B)** Survival curves of all cattle after challenge with a lethal dose of *P. multocida* serogroup B bacteria. Performed as cattle trial 3 at NVI. Serum immunogenicity compared by two-way ANOVA with Dunnett’s multiple comparisons test after each dose against cattle that received adjuvant only. Survival curves compared the PmSLP-3–immunized animals individually against adjuvant only controls with Log-rank (Mantel–Cox) test. Only significant differences are indicated, other comparisons are not significant, * represents p ≤ 0.05, ** represents p ≤ 0.01, and **** represents p ≤ 0.0001.

The serological results in Zebu cattle reflected what was seen in Holstein cattle, where Montanide ISA 61 formulations elicited the highest levels of PmSLP-3–specific antibody and robust titres were seen after only one dose (**Figure 6A**). PmSLP-3 vaccines formulated with either alum-based adjuvant elicited similar titres that required a booster dose to elicit antibodies in all cattle, while neither the bacterin vaccine nor the adjuvant only [Alk(SO_4_)_2_] control elicited PmSLP-3 antibodies, as expected. Three weeks after the booster dose, all cattle were challenged with a lethal dose containing 5.3 × 10^5^ CFU/mL of a capsular type B strain of *P. multocida.* All cattle that received adjuvant alone reached clinical endpoint between 24 h and 48 h after infection, whereas the matched bacterin and all three PmSLP-3 vaccine formulations were fully protective (**Figure 6B**). Of vaccinated animals, only a subset demonstrated any clinical symptoms after infection at approximately 6 h to 18 h after infection: one of the eight cattle in the bacterin group, two of the eight cattle in both alum formulations, and one of the eight cattle of those that received PmSLP-3 formulated with Montanide ISA 61. All vaccinated cattle that exhibited clinical symptoms recovered by 24 h after infection. *P. multocida* was isolated from blood of cattle that displayed clinical symptoms at 12 h to 24 h after infection and from the lung, liver, and spleen of cattle that reached clinical endpoint. Blood samples collected at 10 days after infection from all vaccinated calves were negative for culturable *P. multocida* and were negative by PCR. Additionally, the lungs, liver, and spleen were collected from one surviving animal per vaccine group, and all were negative for culturable *P. multocida* and negative by molecular detection.

### Identifying the number of doses and required immunogen concentration for effective PmSLP-3 vaccination

Due to the unique challenges that LMICs face with maintaining low-cost protein antigen production, it is vital to induce a robust, long-term immune response with a minimal protein dose. Additionally, logistics of vaccine distribution to small-scale livestock owners and cost associated with requiring multiple doses to elicit robust protection make the potential of a single dose vaccine attractive for veterinary products in LMICs. Thus, we next decided to explore optimal protein antigen dose and number of immunizations needed to induce immunogenicity and provide robust clinical protection. Based on the collective results from the adjuvant studies, Montanide ISA 61 was selected for the subsequent studies due to its capabilities of inducing a higher PmSLP-3–specific serum and mucosal antibody response, the maintenance of serum antibody titres over the course of 12 months, and the robust protection seen in cattle trial 3.

**Figure 7:**
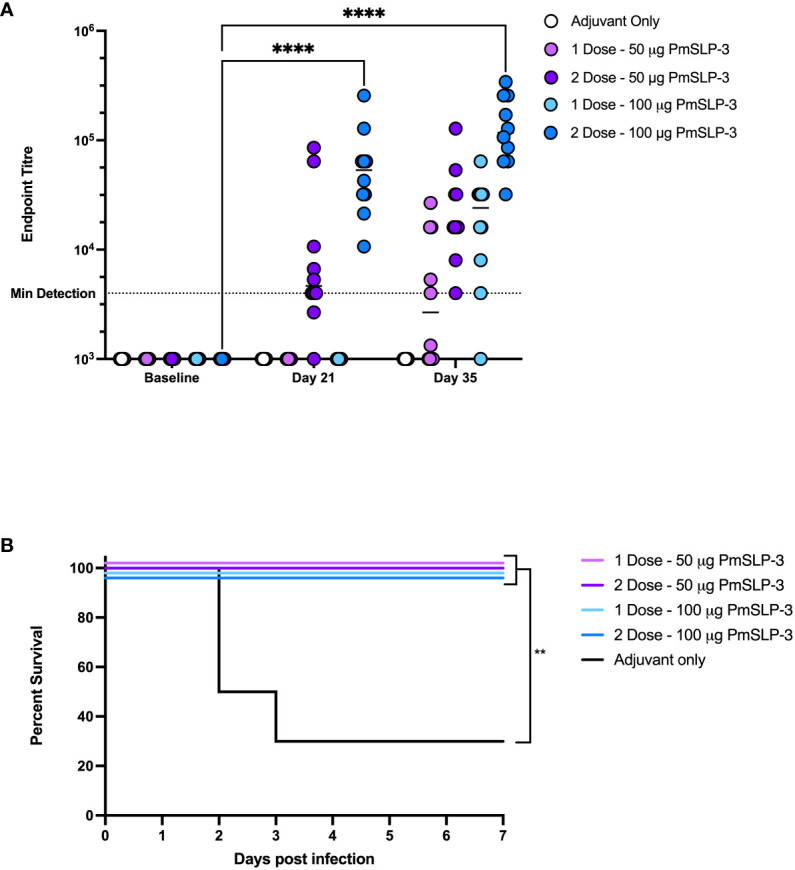
Evaluation of needed immunogen dosing and number of immunizations. **(A)** Immunogenicity of sera from Zebu cattle that received either Montanide ISA 61 only (Adjuvant only, white), or PmSLP-3 formulated with Montanide ISA 61 against purified PmSLP-3 protein. Cattle received either a single dose of 50 µg of PmSLP-3 vaccine (light purple) or 100 µg of PmSLP-3 vaccine (light blue) on day 21, or received two doses of 50 µg of PmSLP-3 vaccine (dark purple) or 100 µg of PmSLP-3 vaccine (dark blue) on day 0 and day 21. Each dot represents the mean of technical triplicates with line representing median. **(B)** Survival curves of all cattle after challenge with a lethal dose of *P. multocida* serogroup B bacteria. Performed as cattle trial 4 at NVI. Serum immunogenicity compared by two-way ANOVA with Dunnett’s multiple comparisons test. Survival curves compared individually against adjuvant only with Log-rank (Mantel–Cox) test. Only significant differences are indicated, other comparisons are not significant, ** represents p ≤ 0.01, and **** represents p ≤ 0.0001.

To establish the minimal dosage required for protection, we performed a blinded challenge trial in 1- to 2-year-old Zebu cattle at NVI (cattle trial 4). Cattle received either one or two doses of formulations containing either 50 µg or 100 µg of PmSLP-3 antigen per dose. Two doses of PmSLP-3 vaccine containing either protein concentration gave a robust and, for the 100-µg dose, significant increase in anti–PmSLP-3 serum antibodies compared to adjuvant alone (**Figure 7A**); however, a single dose of vaccine did not elicit statistically significant titres when given 3 weeks pre-challenge. All animals had local swelling, likely due to the adjuvant included in the formulation being consistent across all groups.

Cattle were then challenged with the serogroup B strain of *P. multocida* as before. All PmSLP-3–containing vaccine formulations elicited robust protection, with all cattle being protected (**Figure 7B**), compared to cattle that received adjuvant only where 70% lethality was observed by 3 days after infection. Cattle that received either one or two doses of vaccine containing 100 µg of PmSLP-3 were fully protected from clinical symptoms, whereas one of the 10 cattle that received either one or two doses of 50 µg of PmSLP-3–containing vaccine displayed symptoms 12 h after infection and recovered by 24 h after infection. All cattle that exhibited clinical symptoms had recoverable *P. multocida* in the blood, and all animals that reached clinical endpoint had recoverable *P. multocida* and detectable bacteria by PCR in the lung, liver, and spleen. Surviving animals had no recoverable bacteria from blood at 10 days after challenge. Necropsy was performed on one animal per group from each vaccine group, where no recoverable *P. multocida* was detected in the lung, liver, or spleen.

**Figure 8:**
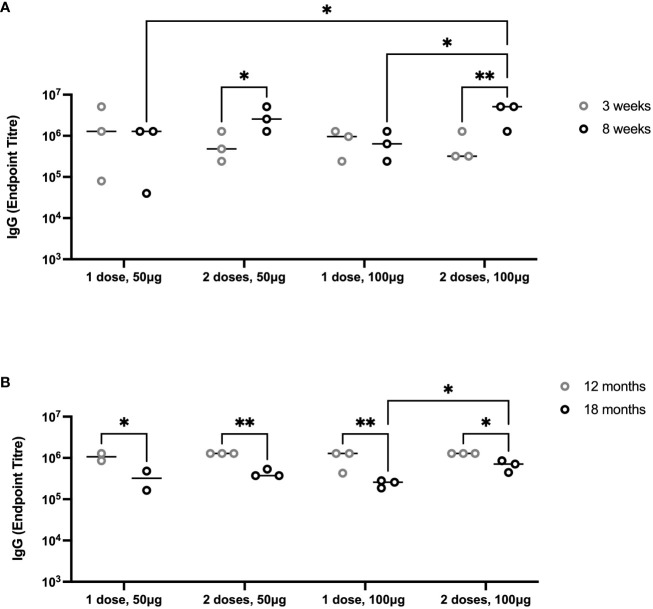
Duration of immunity after immunization with PmSLP-3 antigen. **(A)** Groups of three 6-month-old female Holstein calves were vaccinated i.m. with PmSLP-3 antigen formulated with Montanide ISA 61 either once (day 0) or twice (day 0 and day 21) with either 50 µg or 100 µg of protein antigen. PmSLP-3–specific plasma IgG was evaluated at 3 weeks (gray) and 8 weeks (black) after day 0. **(B)** PmSLP-3–specific plasma IgG was evaluated at 12 months (gray) and 18 months (black) after immunization to evaluate for the duration of immunity. Performed as cattle trial 2 at the University of Calgary. Serology was run on animals in separate experiments due to the long time span between sampling and, therefore, is plotted separately. Plasma immunogenicity compared by two-way repeated-measures ANOVA with Uncorrected Fisher’s LSD multiple comparisons test. Line represents median. Only significant differences are indicated, other comparisons are not significant, * represents p ≤ 0.05, and ** represents p ≤ 0.01.

To evaluate the durability of lower doses of immunogen and number of vaccinations, we immunized groups of 6-month-old female Holstein calves (n = 3 per group) with one or two doses of 50 µg or 100 µg of PmSLP-3 antigen, respectively (cattle trial 2). Due to the local reactogenicity of water-in-oil adjuvants, including Montanide ISA 61, we performed vaccination intra-muscularly instead of the previously used sub-cutaneous route and observed less local swelling after vaccination. Consistent with the observations in Zebu cattle, both protein doses were immunogenic after one dose in Holstein calves, and performing a second immunization significantly increased the anti–PmSLP-3 plasma titres (**Figure 8A**). Plasma was sampled at 12 and 18 months after immunization, where anti–PmSLP-3 plasma titres were maintained at both time points, although there was a reduction in all groups at 18 months after immunization, indicating a gradual waning (**Figure 8B**).

### Evaluating the duration of protection from PmSLP-3 vaccination

Vaccination of cattle with PmSLP-3 formulated with Montanide ISA 61 is not only able to induce a high serum antibody response but also to maintain prolonged serum antibodies over a minimum of 3 years. This is promising considering the highly seasonal and cyclic outbreaks of HS in East Africa and South Asia. To assess how long protective efficacy is maintained after a single dose of PmSLP-3–containing vaccine, 1- to 2-year-old Zebu cattle (n = 10 per group for PmSLP-3– or bacterin-vaccinated cattle, n = 8 for naïve cattle) were vaccinated sub-cutaneously and challenged 31 weeks later (cattle trial 5). As a subset of cattle that received vaccine containing 50 µg of PmSLP-3 exhibited symptoms after challenge in cattle trial 4 and elicited lower antibody levels after immunization, we opted to evaluate the 100-µg formulation for the duration of protection study. Vaccine was again formulated with Montanide ISA 61. 2/10 cattle that received the local bacterin displayed swelling at the injection site, whereas eight of the 10 cattle that received PmSLP-3 vaccine had local reactions with no major reactions occurring.

**Figure 9:**
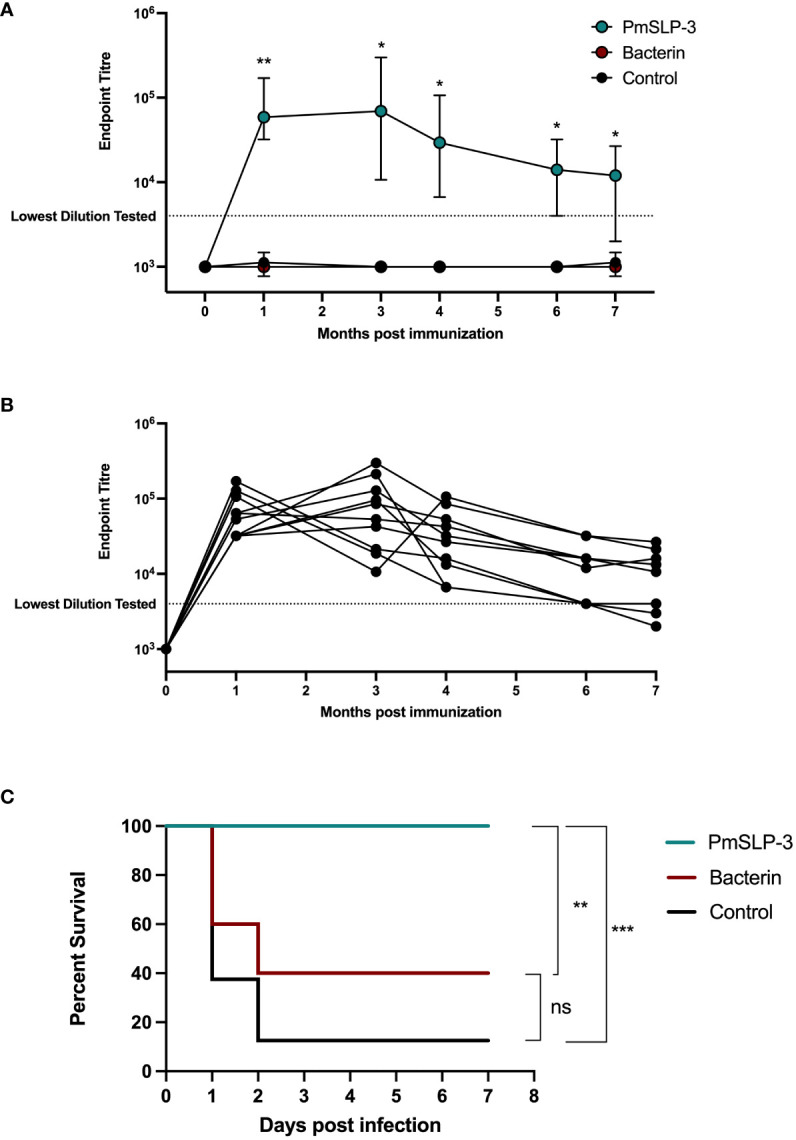
Evaluation of duration of immunity by PmSLP-3 formulated with Montanide ISA 61. **(A)** Immunogenicity of Zebu cattle immunized with PmSLP-3 (teal), the local bacterin (red), or control (naïve) animals (black). PmSLP-3–specific serum IgG was evaluated prior to immunization as well as at 1 month, 3 months, 4 months, 6 months, and 7 months after vaccination. Data represent median and range of 8 to 10 animals per group. **(B)** PmSLP-3–specific serum IgG from individual cattle immunized with PmSLP-3. **(C)** Survival curves of all cattle after challenge with a lethal dose of *P. multocida* serogroup B bacteria 7 months after immunization. Performed as cattle trial 5 at NVI. Serum samples were tested in technical triplicate, and endpoint titres were compared by two-way repeated-measures ANOVA with *post-hoc* analysis by Tukey’s multiple comparisons test at each time point. Survival curves compared individually with Log-rank (Mantel–Cox) test. ns represents non-significant, * represents p ≤ 0.05, ** represents p ≤ 0.01, and *** represents p ≤ 0.001.

Serum IgG was evaluated for PmSLP-3–specific antibodies at 1 month, 2 months, 4 months, 6 months, and 7 months after cattle received the single dose of vaccine. As expected, all cattle that received PmSLP-3 vaccine elicited robust titres by 1 month after vaccine (**Figures 9A, B**). PmSLP-3–specific antibody persisted in all cattle for at least 4 months after vaccination (**Figure 9B**), with most animals maintaining these titres at 7 months after vaccination.

Upon challenge with 4.5 × 10^5^ CFU of the serogroup B strain of *P. multocida*, seven of the eight naïve (non-vaccinated) cattle reached clinical endpoint by 48 h after infection (**Figure 9C**), and all eight exhibited clinical symptoms. All cattle (10 of 10) that received the local bacterin also displayed clinical symptoms after infection, and only four of 10 survived the challenge, whereas the remaining six reached clinical endpoint by 48 h after infection, and there was no statistical difference in survival between the bacterin-immunized and naïve cattle. Strikingly, all 10 cattle that received PmSLP-3 vaccine survived the challenge, and only two displayed raised rectal temperature and depression at 6 h after infection that resolved by 24 h. Again, all cattle that displayed clinical symptoms had recoverable *P. multocida* in their blood, and all cattle that reached clinical endpoint had recoverable bacteria in the lung, liver, and spleen samples. Cattle that were protected from infection had no recoverable bacteria or bacteria detectable by PCR at 10 days after infection. Of surviving cattle, one per group was sent to necropsy where no *P. multocida* was recovered or detected by PCR from the lung, liver, and spleen samples.

### Evaluating the ability of PmSLP-3 vaccination to protect from serogroup E strains of *P. multocida*

**Figure 10:**
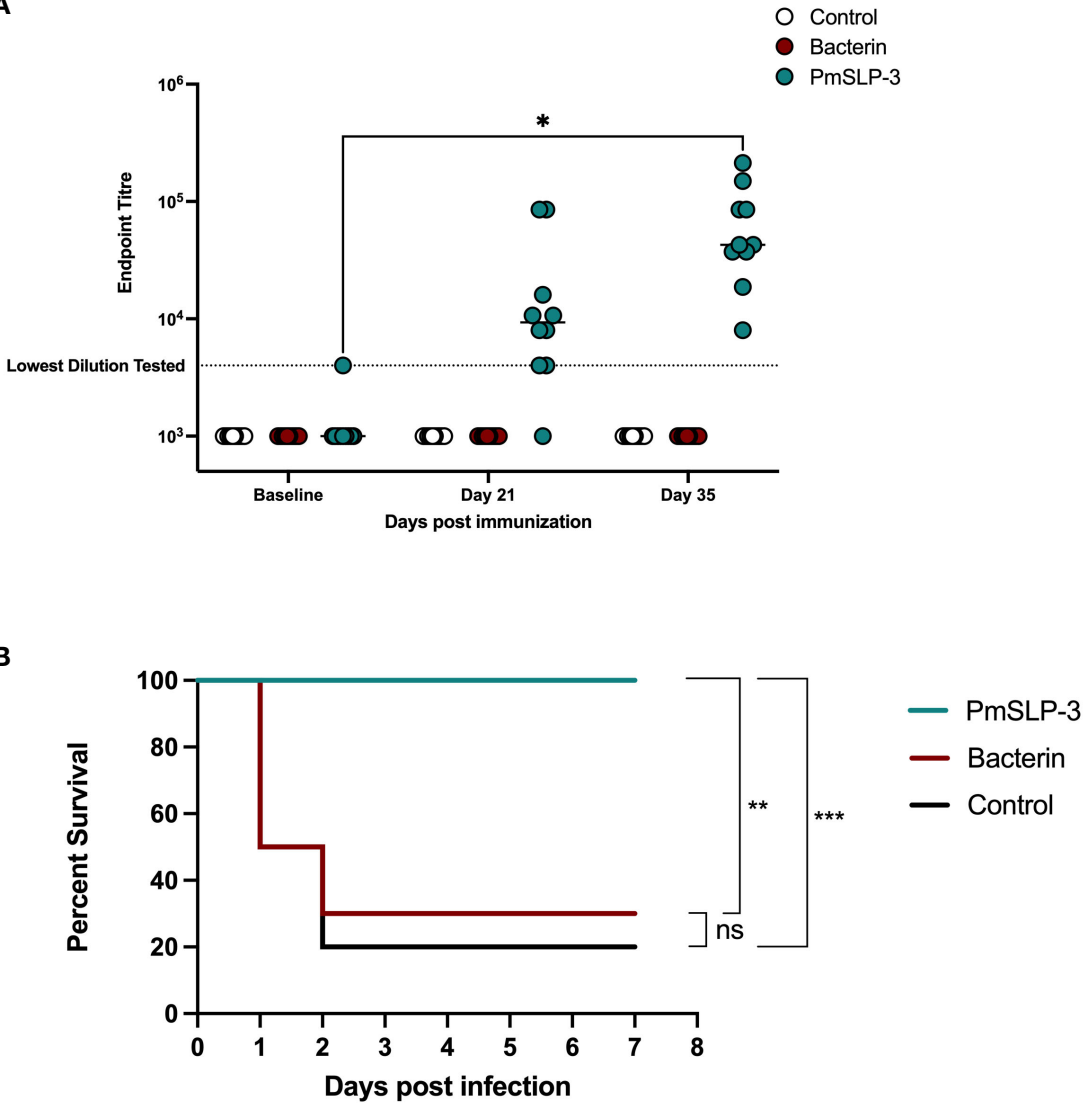
Evaluation of protection against a serogroup E strain of *P. multocida* by a single dose of PmSLP-3 vaccine formulated with Montanide ISA 61. **(A)** Immunogenicity of sera from Zebu cattle that received either PmSLP-3 formulated with Montanide ISA 61 (teal), the local bacterin (red), or control (naïve) animals (white) tested against purified PmSLP-3 protein. Each dot represents the mean of technical triplicates and line represents median. **(B)** Survival curves of all cattle after challenge with a lethal dose of *P. multocida* serogroup E bacteria. Performed as cattle trial 6 at NVI. PmSLP-3 titres tested by two-way repeated-measures ANOVA with Tukey’s multiple comparisons test with titres compared to baseline. Survival curves compared individually against adjuvant only with Log-rank (Mantel–Cox) test. Only significant differences are noted forl **(A)**, non-significant differences are not shown. ns represents non significant, * represents p ≤ 0.05, ** represents p ≤ 0.01, and *** represents p ≤ 0.001.

HS is also caused by serogroup E strains of *P. multocida*; therefore, we sought to evaluate PmSLP-3–mediated protection against a serogroup E capsulated strain. One- to 2-year-old Zebu cattle (n = 10 per group) were immunized either twice with the local bacterin (originating from a capsule B strain) or once with PmSLP-3 formulated with Montanide ISA 61 via sub-cutaneous injection (cattle trial 6). Local swelling after immunization was noted in one of the 10 cattle that received the bacterin and six of the 10 cattle that received the PmSLP-3 formulation. As expected, cattle that received PmSLP-3 vaccine elicited PmSLP-3–specific serum IgG (**Figure 10A**), which was not seen in naïve or bacterin immunized animals.

Five weeks after immunization, cattle were challenged with 6.5 × 10^5^ CFU of a serogroup E strain of *P. multocida*. All cattle that received either the bacterin vaccine or were in the naïve cohort exhibited clinical symptoms, with seven of the 10 cattle and eight of the 10 cattle reaching clinical endpoint by 48 h after infection, respectively (**Figure 10B**). In contrast, all cattle that received a single dose of PmSLP-3 vaccine survived the course of infection, and only three cattle displayed clinical symptoms, which included raised rectal temperature and a dull disposition at 12 h after infection that resolved by 24 h. Consistent with previous cattle challenge studies, *P. multocida* was recovered from the blood of animals exhibiting clinical symptoms, as well as from the lung, liver, and spleen samples from animals that reached endpoint. Blood samples taken at 10 days after infection were negative for *P. multocida* by PCR and culture negative. Necropsy samples were taken from one animal per vaccine group from those that survived the infection, and all were negative for *P. multocida* re-isolation and PCR detection.

## Discussion

Infections with *P. multocida* can cause devastating infections, particularly in LMICs where strains causing HS are endemic, and where economic losses associated with animal mortality are particularly harmful to small holder farmers. Prevention of these infections by efficacious and affordable vaccines is needed; however, current bacterin vaccines offer serogroup specific protection of limited duration and may be highly reactogenic due to the high dose of inflammatory agonists from the inactivated bacteria present in these vaccines. Therefore, here we have evaluated novel protein-based vaccines targeting the bacterial surface lipoprotein, PmSLP-3.

We selected a variety of traditional adjuvants (aluminum hydroxide gel and aluminum potassium sulfate) as well as some newer veterinary adjuvants with differing characteristics (Emulsigen-D, Montanide Gel 02, Montanide ISA 61) for inclusion in the studies presented here, which aim toward identifying the most efficacious vaccine formulation. Alum salts predominantly induce a T_H_2 response and improve targeting of antigens to antigen presenting cells (APCs). The alum-based adjuvants were assessed as these are the predominant adjuvant used in veterinary vaccines, have a robust safety profile, and are easy to formulate. However, bacterin vaccines for HS adjuvanted with aluminum potassium sulfate have the disadvantage of weak immunity of short duration and lack of stability (27).

Montanide Gel 02 is a polymer-based adjuvant where gel particles of sodium polyacrylate are dispersed in water to obtain a depot effect, aimed at increasing innate immune cell recruitment, and this adjuvant has been previously shown to be safe in multiple species including cattle (28). Here, we tested Montanide Gel 02 both with and without the TLR-3 agonist Poly(I:C), as our original studies used this combined formulation when establishing that PmSLP-based immunization can protect from *P. multocida* infection. Emulsigen-D is a water-in-oil adjuvant containing the immunostimulant dimethyldioctadecyl ammonium bromide (DDA), which has been previously shown to have more robust immunostimulatory properties in a DNA vaccine for rabies in BALB/c mice when compared to Emulsigen without DDA or in non-adjuvanted vaccines (29). Both Montanide Gel 02 and Emulsigen-D are easy to formulate with antigen by gentle agitation, an attractive feature for use in LMICs, particularly with an antigen such as PmSLP that has been previously shown to be efficacious after lyophilization (21). Montanide ISA 61 is a water-in-oil based adjuvant specifically formulated for veterinary use in bovines, utilizing mineral oil that has been noted to elicit long-term immune responses. In contrast to the other adjuvants used, formulation with Montanide ISA 61 requires high shear to make a stable emulsion. Montanide ISA 61 is widely used in cattle, including in a foot-and-mouth disease–inactivated viral vaccine with robust immunogenicity (30), a viral recombinant protein vaccine for BVDV E2 glycoprotein where Montanide ISA 61 induced greater protection in mice than recombinant glycoprotein alone (31), as well as a recombinant protein vaccine for bacteria, where a formulation of recombinant *Mycoplasma mycoides* (CBPP) protein with Montanide ISA 61 showed 81% protection compared to 22% from attenuated live vaccine (32). In sheep, Montanide ISA 61 showed protection against the liver fluke *Fasciola hepatica* where the formulation containing Montanide ISA 61 outperformed the formulation with aluminum hydroxide gel (33).

When evaluating different formulations, we put emphasis on minimizing the number of doses of vaccine required and increasing the duration of immunity to increase the accessibility of a final vaccine product. Early studies in mice that informed the selection of adjuvant candidates in cattle showed that formulations containing both Montanide Gel 02 + Poly(I:C) and Montanide ISA 61 were highly efficacious and elicited high levels of PmSLP-1-specific serum IgG and its subclasses (**Figure 1**). These titres were maintained for a minimum of 6 months (**Figure 2**). In contrast, when we evaluated immunogenicity in Holstein cattle, PmSLP-3 formulated with Montanide ISA 61 elicited significantly higher levels of PmSLP-3–specific antibodies than did the gel-based formulation (**Figure 3**). Our previous studies heavily utilized challenge studies in mice to identify PmSLP-mediated protection, and these were predictive of the protection that we observed in cattle (21). In some jurisdictions, mouse studies are also required for regulatory approval of killed *P. multocida* vaccines (as part of the American Code of Federal Regulations 9CFR§113.121). Here, we saw that several adjuvant formulations with PmSLP elicited robust and long-lasting titres in mice; however, in cattle, Montanide ISA 61 with PmSLP-3 elicited clearly higher titres. This indicates that, while mice are a robust model for vaccine antigen selection and useful to minimize the number of formulations that need to be tested in larger animals, trials in the target animal are vital for selecting the optimal formulation.

With our chosen formulation of PmSLP-3 with Montanide ISA 61, immunized Holstein cattle maintained robust antibody titres for at least 3 years after immunization (**Figure 4E**). It also elicited the highest level of PmSLP-3–specific serum IgG1 and IgG2 (**Figures 3B, C**), antibody subclasses that are thought to be involved with bacterial clearance via complement-dependent cytotoxicity (IgG1) and neutrophil and macrophage binding (IgG2) (26). This formulation also provided the highest levels of mucosal PmSLP-3–specific IgG (**Figure 5**); as mucosal antibodies have the potential to reduce both carriage and disease caused by *P. multocida*, this was considered particularly beneficial. The nasopharyngeal mucosa is the normal site of colonization of *P. multocida* (34), and, indeed, this is why other candidate HS vaccines have considered intranasal immunization (18, 35) and immunization with aerosolized attenuated live vaccines (13, 27) to better prevent infections. Contrary to the gastrointestinal tract, secreted IgGs (sIgGs) are considered to play an important role in the mucosal defense of the nasopharyngeal associated lymphoid tissue of cattle (36). The induction of consistently high mucosal IgG after immunization with PmSLP-3 vaccine formulated with Montanide ISA 61 is, therefore, very encouraging. However, transmission of *P. multocida* occurs mainly through carrier animals where bacteria are harbored in the tonsillary crypts (8), where sIgGs may not impact clearance. It is, therefore, imperative to avoid generating carrier animals by only partial vaccine protection.

Subsequent analysis of PmSLP-3 with Montanide ISA 61 in Zebu cattle showed highly consistent serological data as were seen with Holstein cattle (**Figures 3A**, **6A**), suggesting that cattle breed does not substantially alter the immunological response to PmSLP-3–based vaccines and thus broadening the potential impact of the results shown here. While we have not included buffalo in our studies, based on the studies conducted with OmpH, buffaloes show similar immunologic responses (18). Thus, we are hopeful that the protective efficacy demonstrated here in cattle will also extend to buffaloes.

The dosing studies performed here aimed to evaluate whether a prime-boost vaccination schedule was required to elicit an effective immune response or whether a single dose is sufficient. While two doses of vaccine did elicit higher antibodies compared to a single dose (**Figures 7A**, **8A**), there was no difference in protective efficacy (**Figure 7B**) or maintenance of serum antibodies (**Figure 8B**). Indeed, antigen dose also did not play a substantial role, as both 50 µg and 100 µg of PmSLP-3 elicited full protection from challenge (**Figure 7B**). The 100 µg of PmSLP-3 formulation was selected to go forward for our latter studies because a small subset of cattle that received only 50 µg showed symptoms after *P. multocida* challenge, an outcome that was absent in all cattle that received the higher dose. When Zebu cattle were challenged 7 months after a single dose of 100 µg of PmSLP-3 vaccine formulated with Montanide ISA 61, all cattle were fully protected, a striking outcome that was not accomplished with the local matched bacterin vaccine (**Figure 9**). These data together indicate that PmSLP-3 vaccines can elicit a superior duration of immunity compared to currently available vaccines. That protection was extended to a serogroup E challenge strain that again was not achieved with the serogroup B–derived bacterin (**Figure 10**), providing an additional benefit of PmSLP-3 vaccination. PmSLP-3 sequences are highly conserved among HS-causing strains; therefore, it was expected that protection would extend to both capsule type B– and type E–containing strains.

The selection of PmSLP-3 formulated with Montanide ISA 61 has led to a highly immunogenic and protective vaccine; however, we did note high levels of reactogenicity from this formulation in both mice (sub-cutaneous administration) as well as cattle (sub-cutaneous or intra-muscular administration). While we have not compared route of immunization side by side, less visible swelling was noted in cattle that received intra-muscular injection of PmSLP-3 with Montanide ISA 61, which may affect the decision of which route to pursue for licensure in the future. It should be noted that, even with sub-cutaneous administration, reactions were limited to after immunization swelling, and no major impact was seen on animal wellbeing. Additionally, the need for high shear with Montanide ISA 61 to produce a stable emulsion may mean that accessibility of this vaccine is lower than using adjuvants that require basic mixing where lyophilized antigen can be reconstituted by the end user. However, the benefit of a one-dose vaccine was prioritized as more important for accessibility for an HS vaccine. Important in this regard, the vaccine used for our duration of protection study (**Figure 9**) and serogroup E study (**Figure 10**) was utilized approximately 6 months and 12 months after formulation and storage at 4°C, demonstrating the shelf stability of this highly effective formulation.

Both the regulatory landscape and the availability and accessibility of veterinary services, including the implementation of prophylactic vaccination programs, vary greatly among affected LMICs (7, 37). Vaccine coverage is low due to lack of knowledge about vaccine programs and challenges in vaccine distribution to extensively kept livestock herds, where animals are maintained in grazing fields generally at a lower density compared to “intensive” indoor systems where animals are maintained at higher density with more interventions. According to the World Organization for Animal Health (previously the Office International des Epizooties), this is dependent on multiple factors, resulting in a lack of a coherent vaccine strategy and an inability to reach 80% vaccine coverage needed to prevent large HS outbreaks. Standard procedures include the seasonal use of local bacterin or oil adjuvanted vaccines (38, 39). Currently used oil adjuvanted vaccines are administered 1 month before monsoon season and show a duration of immunity of up to 1 year after booster immunization. However, applying high viscosity fluids and, in particular, gathering animals for a booster vaccination is challenging. Additionally, livestock owners are concerned with local reactions at the injection site and the impact on meat quality. Given the long duration of immunity, the protective coverage of both serotypes, and the possibility of full, lasting protection after only one vaccination, we therefore see a great potential for PmSLP-3 as a viable vaccine antigen for prevention of HS in LMIC applications.

BRD-causing strains of *P. multocida* analyzed to date do not contain a PmSLP-3 antigen, and we have previously shown that there is no cross-protection elicited between PmSLP-1 and PmSLP-3 (21). Therefore, additional work needs to be done to develop bovine vaccines able to protect against both HS- and BRD-causing strains of *P. multocida*. Vaccines containing multiple PmSLPs may be able to extend coverage to both disease types; however, the geographic distribution of infection types may mean that such vaccines are of limited interest when considering the additional cost of including multiple immunogens. Recent HS outbreaks in Western Europe indicate that disease distribution may be changing (6), and, therefore, the potential impact of a *P. multocida* vaccine able to target both HS and BRD in cattle may become more useful.

Together, our findings demonstrate that PmSLP-3 formulated with Montanide ISA 61 is an effective vaccine that elicits robust antibody responses that persisted for at least 3 years, can elicit protection with a single dose, and is able to protect against both serogroup B and E strains of *P. multocida.*

## Data availability statement

The original contributions presented in the study are included in the article/supplementary material. Further inquiries can be directed to the corresponding authors.

## Ethics statement

Mouse studies were performed under animal use protocol 20011319 which was approved by the animal care committee at the University of Toronto, Canada. Cattle studies were performed under animal use protocol AC20-0007 at the University of Calgary, Canada, and under local regulatory approval at the National Veterinary Institute (NVI) in Ethiopia. The studies were conducted in accordance with the local legislation and institutional requirements.

## Author contributions

JF: Conceptualization, Data curation, Formal analysis, Writing – original draft, Writing – review & editing. RW: Conceptualization, Writing – review & editing, Data curation, Formal analysis, Writing – original draft. EI: Conceptualization, Data curation, Formal analysis, Validation, Writing – review & editing. LT: Data curation, Writing – review & editing. GD: Data curation, Writing – review & editing. DD: Data curation, Writing – review & editing. EA: Data curation, Writing – review & editing. WW: Data curation, Writing – review & editing. AL: Data curation, Writing – review & editing. MA: Data curation, Writing – review & editing. BB: Data curation, Writing – review & editing. QN: Data curation, Writing – review & editing. DN: Data curation, Writing – review & editing. SA: Data curation, Writing – review & editing. AS: Writing – review & editing. TT: Writing – review & editing. TM: Conceptualization, Supervision, Writing – review & editing. SG-O: Writing – review & editing.
